# The genome sequence of the Green-brindled Crescent, *Allophyes oxyacanthae* (Linnaeus, 1758)

**DOI:** 10.12688/wellcomeopenres.18935.1

**Published:** 2023-02-03

**Authors:** Douglas Boyes, University of Oxford and Wytham Woods Genome Acquisition Lab, Darwin Tree of Life Barcoding collective, Wellcome Sanger Institute Tree of Life programme, Wellcome Sanger Institute Scientific Operations: DNA Pipelines collective, Tree of Life Core Informatics collective, Peter W. H. Holland, Darwin Tree of Life Consortium

**Affiliations:** 1 UK Centre for Ecology and Hydrology, Wallingford, Oxfordshire, UK; 2 University of Oxford, Oxford, Oxfordshire, UK

**Keywords:** Allophyes oxyacanthae, Green-brindled Crescent, genome sequence, chromosomal, Lepidoptera

## Abstract

We present a genome assembly from an individual male *Allophyes oxyacanthae* (the Green-brindled Crescent; Arthropoda; Insecta; Lepidoptera; Noctuidae). The genome sequence is 458 megabases in span. The whole assembly is scaffolded into 31 chromosomal pseudomolecules, including the assembled Z sex chromosome. The mitochondrial genome has also been assembled and is 15.3 kilobases in length. Gene annotation of this assembly on Ensembl has identified 17,301 protein coding genes.

## Species taxonomy

Eukaryota; Metazoa; Ecdysozoa; Arthropoda; Hexapoda; Insecta; Pterygota; Neoptera; Endopterygota; Lepidoptera; Glossata; Ditrysia; Noctuoidea; Noctuidae; Amphipyrinae; *Allophyes*; *Allophyes oxyacanthae* (Linnaeus, 1758) (NCBI:txid988056]).

## Background

The Green-brindled Crescent *Allophyes oxyacanthae* is a moth in the family Noctuidae with an autumn flight period in the UK. The typical form of the moth is unmistakable, with a dense scattering of shimmering metallic green scales on the forewings, contrasting against a deep brown ground colour, pale marginal band and pale orbicular and reniform stigmata (oval and kidney marks). A distinct colour variant is also encountered in the UK, denoted form *capucina*, with uniform brown colouration and fewer green scales. Breeding experiments suggest that the difference between the two forms is controlled by a single genetic locus, with the f. *capucina* allele dominant to the wild type allele ( Steward, 1977). It has been suggested that f. *capucina* moths may have had a selective advantage in areas of industrial pollution, although the data are unclear on this issue ( Ford, 1967). The genetic locus and molecular basis of the polymorphism have not been identified.

*A. oxyacanthae* has been recorded across most of UK, including the north of Scotland, through Scandinavia and across mainland Europe although there are few records from Italy and southern Spain ( GBIF Secretariat, 2022). The species is attracted to light and can be found in woodlands and gardens where the larval food plants of hawthorn, blackthorn, rowan and fruit trees are found ( Robinson *et al.*, 2010).

A genome sequence for *A. oxyacanthae* will facilitate study of the genetic basis of colour polymorphism and the molecular adaptations underpinning polyphagy, and contribute to a growing data set of resources for understanding lepidopteran biology.

## Genome sequence report

**Figure 1.**
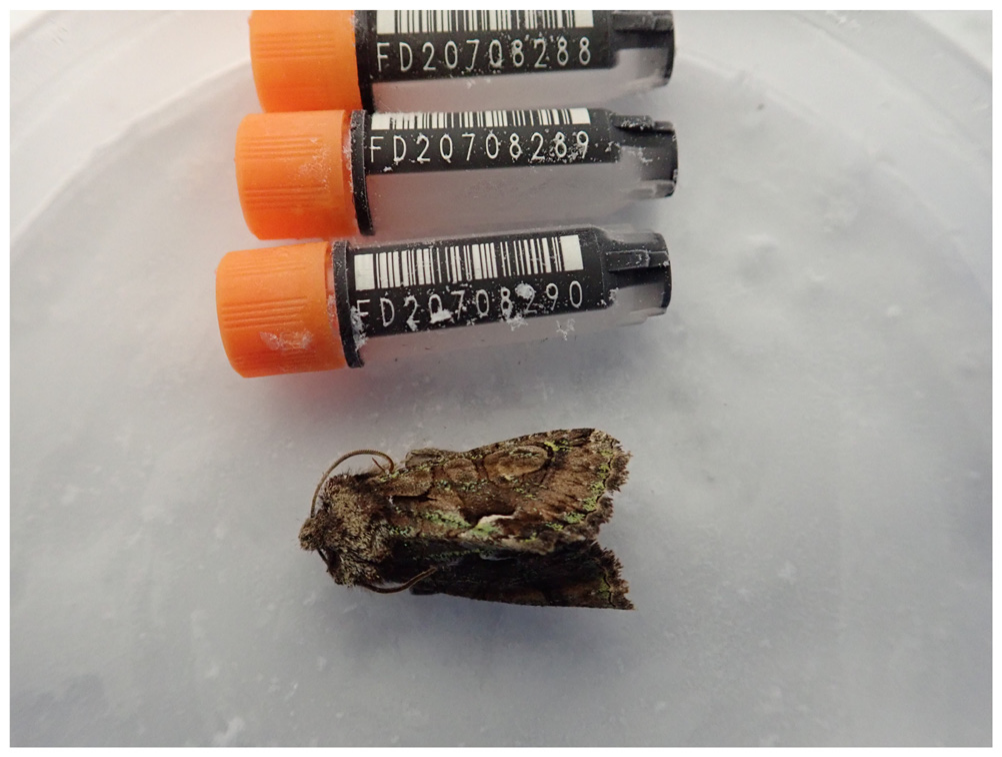
Photograph of the *Allophyes oxyacanthae* (ilAllOxya1) specimen used for genome sequencing.

The genome was sequenced from one male *Allophyes oxyacanthae* specimen ( Figure 1) collected in Wytham Woods (latitude 51.77, longitude –1.34). A total of 71-fold coverage in Pacific Biosciences single-molecule HiFi long reads weas generated. Primary assembly contigs were scaffolded with chromosome conformation Hi-C data. Manual assembly curation corrected three missing or mis-joins, reducing the scaffold number by 8.82%. 

**Figure 2.**
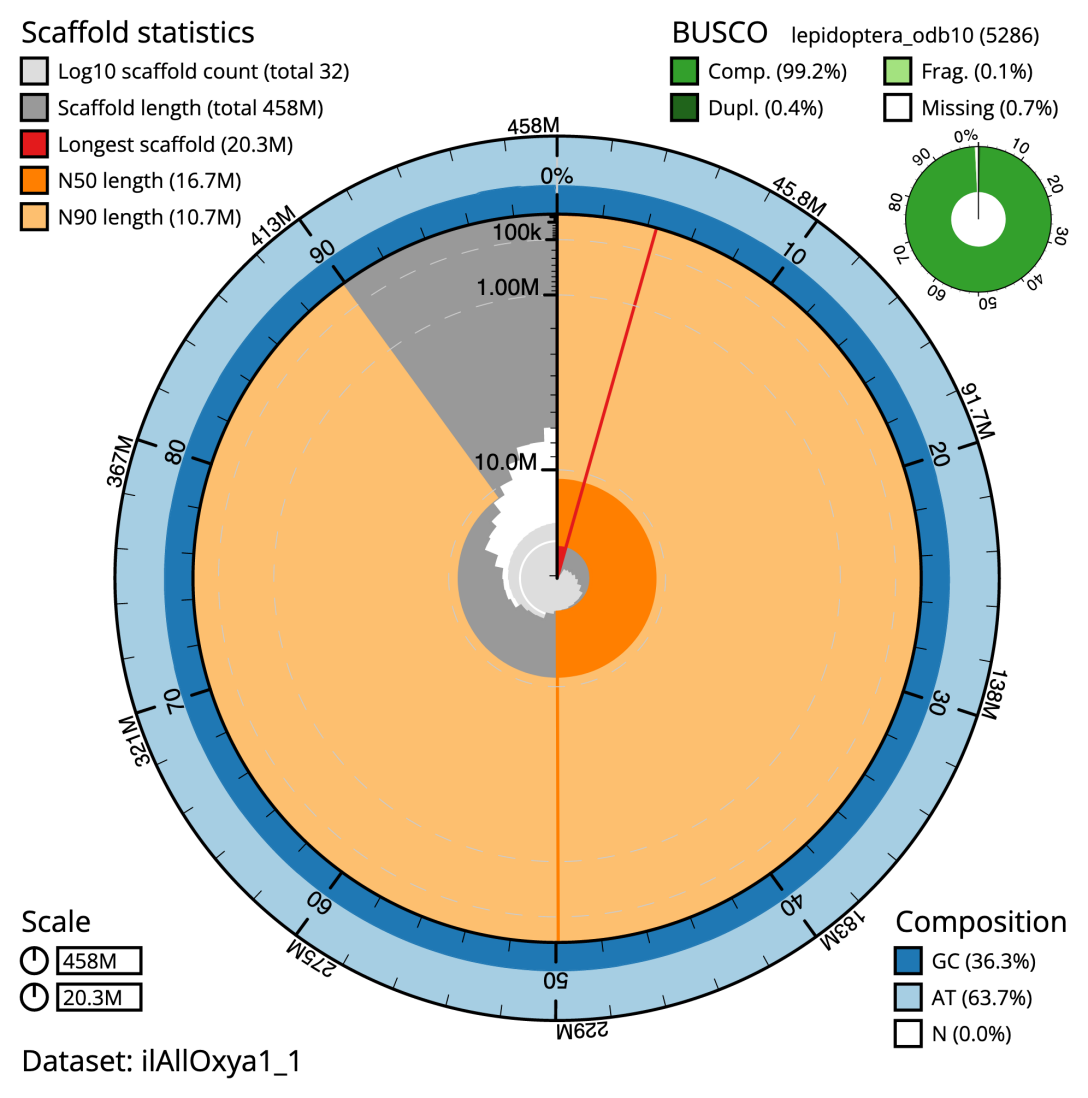
Genome assembly of *Allophyes oxyacanthae*, ilAllOxya1.1: metrics. The BlobToolKit Snailplot shows N50 metrics and BUSCO gene completeness. The main plot is divided into 1,000 size-ordered bins around the circumference with each bin representing 0.1% of the 458,479,537 bp assembly. The distribution of scaffold lengths is shown in dark grey with the plot radius scaled to the longest scaffold present in the assembly (20,278,968 bp, shown in red). Orange and pale-orange arcs show the N50 and N90 scaffold lengths (16,683,655 and 10,717,387 bp), respectively. The pale grey spiral shows the cumulative scaffold count on a log scale with white scale lines showing successive orders of magnitude. The blue and pale-blue area around the outside of the plot shows the distribution of GC, AT and N percentages in the same bins as the inner plot. A summary of complete, fragmented, duplicated and missing BUSCO genes in the lepidoptera_odb10 set is shown in the top right. An interactive version of this figure is available at https://blobtoolkit.genomehubs.org/view/ilAllOxya1_1/dataset/ilAllOxya1_1/snail.

The final assembly has a total length of 458.5 Mb in 31 sequence scaffolds with a scaffold N50 of 16.7 Mb ( Table 1). The whole assembly sequence was assigned to 31 chromosomal-level scaffolds, representing 30 autosomes and the Z sex chromosome. Chromosome-scale scaffolds confirmed by the Hi-C data have been named in order of size ( Figure 2– Figure 5; Table 2). The assembly has a BUSCO v5.3.2 ( Manni *et al.*, 2021) completeness of 99.2% (single 98.8%, duplicated 0.4%) using the lepidoptera_odb10 reference set ( *n* = 5286). While not fully phased, the assembly deposited is of one haplotype. Contigs corresponding to the second haplotype have also been deposited.

**Table 1.** Genome data for *Allophyes oxyacanthae*, ilAllOxya1.1.

Project accession data		
Assembly identifier	ilAllOxya1.1	
Species	*Allophyes oxyacanthae*	
Specimen	ilAllOxya1	
NCBI taxonomy ID	988056	
BioProject	PRJEB50741	
BioSample ID	SAMEA8603204	
Isolate information	ilAllOxya1; male: thorax (PacBio), head (Hi-C)	
Assembly metrics *		*Benchmark*
Consensus quality (QV)	67.9	*≥ 50*
*k*-mer completeness	100%	*≥ 95%*
BUSCO **	C:99.2%[S:98.8%,D:0.4%], F:0.1%,M:0.7%,n:5,286	*C ≥ 95%*
Percentage of assembly mapped to chromosomes	100%	*≥ 95%*
Sex chromosomes	Z chromosome	*localised homologous pairs*
Organelles	Mitochondrial genome assembled	*complete single alleles*
Raw data accessions		
PacificBiosciences SEQUEL II	ERR8575376, ERR8575377	
Hi-C Illumina	ERR8571663	
Genome assembly		
Assembly accession	GCA_932294325.1	
*Accession of alternate haplotype*	GCA_932294395.1	
Span (Mb)	458.5	
Number of contigs	38	
Contig N50 length (Mb)	16.4	
Number of scaffolds	31	
Scaffold N50 length (Mb)	16.7	
Longest scaffold (Mb)	20.3	
Genome annotation		
Number of protein-coding genes	17,301	
Number of transcripts	17,485	

* Assembly metric benchmarks are adapted from column VGP-2020 of “Table 1: Proposed standards and metrics for defining genome assembly quality” from ( Rhie *et al.*, 2021). ** BUSCO scores based on the lepidoptera_odb10 BUSCO set using v5.3.2. C = complete [S = single copy, D = duplicated], F = fragmented, M = missing, n = number of orthologues in comparison. A full set of BUSCO scores is available at https://blobtoolkit.genomehubs.org/view/ilAllOxya1_1/dataset/ilAllOxya1_1/busco.

**Figure 3.**
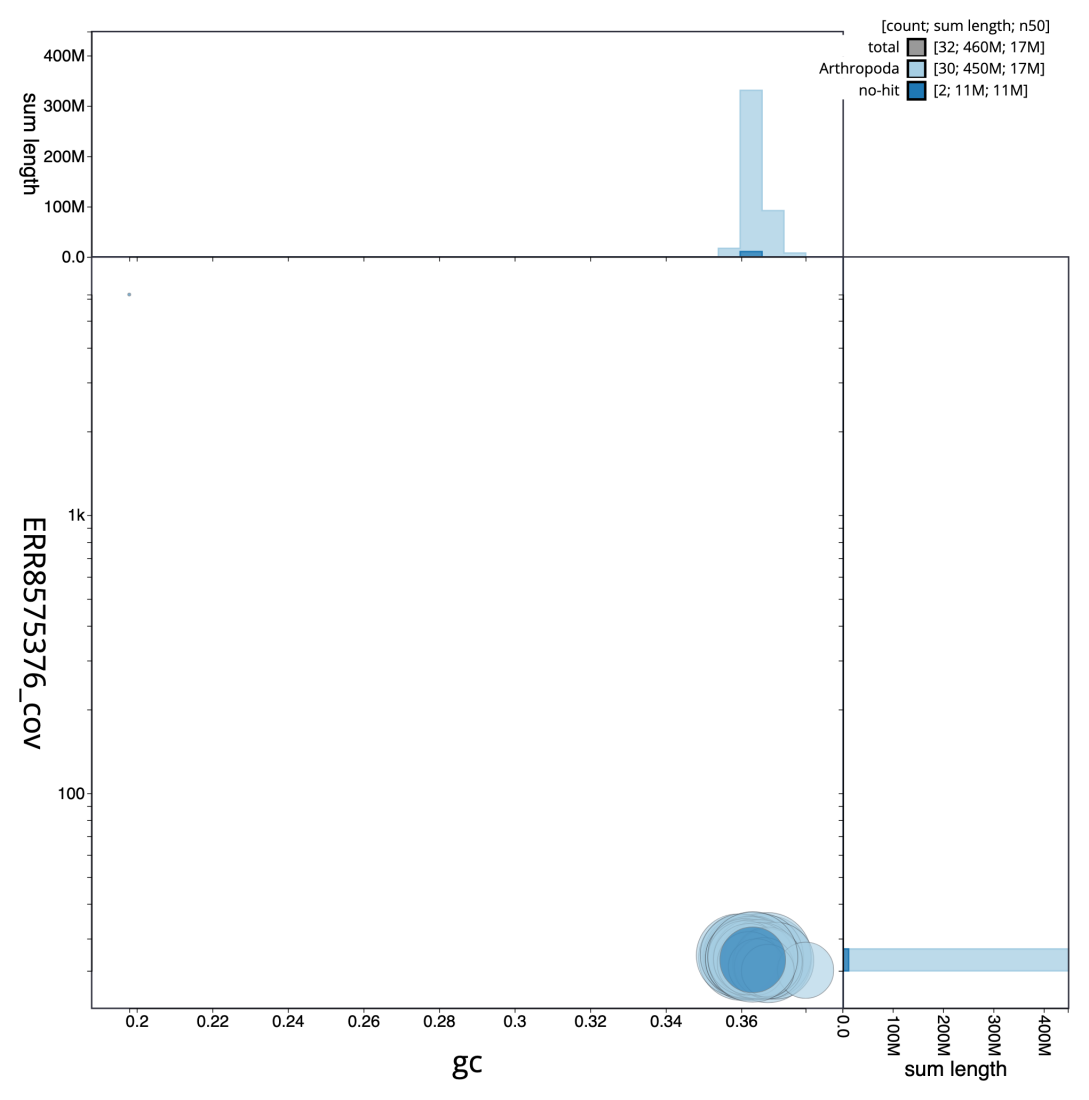
Genome assembly of *Allophyes oxyacanthae*, ilAllOxya1.1: GC coverage. BlobToolKit GC-coverage plot. Scaffolds are coloured by phylum. Circles are sized in proportion to scaffold length. Histograms show the distribution of scaffold length sum along each axis. An interactive version of this figure is available at https://blobtoolkit.genomehubs.org/view/ilAllOxya1_1/dataset/ilAllOxya1_1/blob.

**Figure 4.**
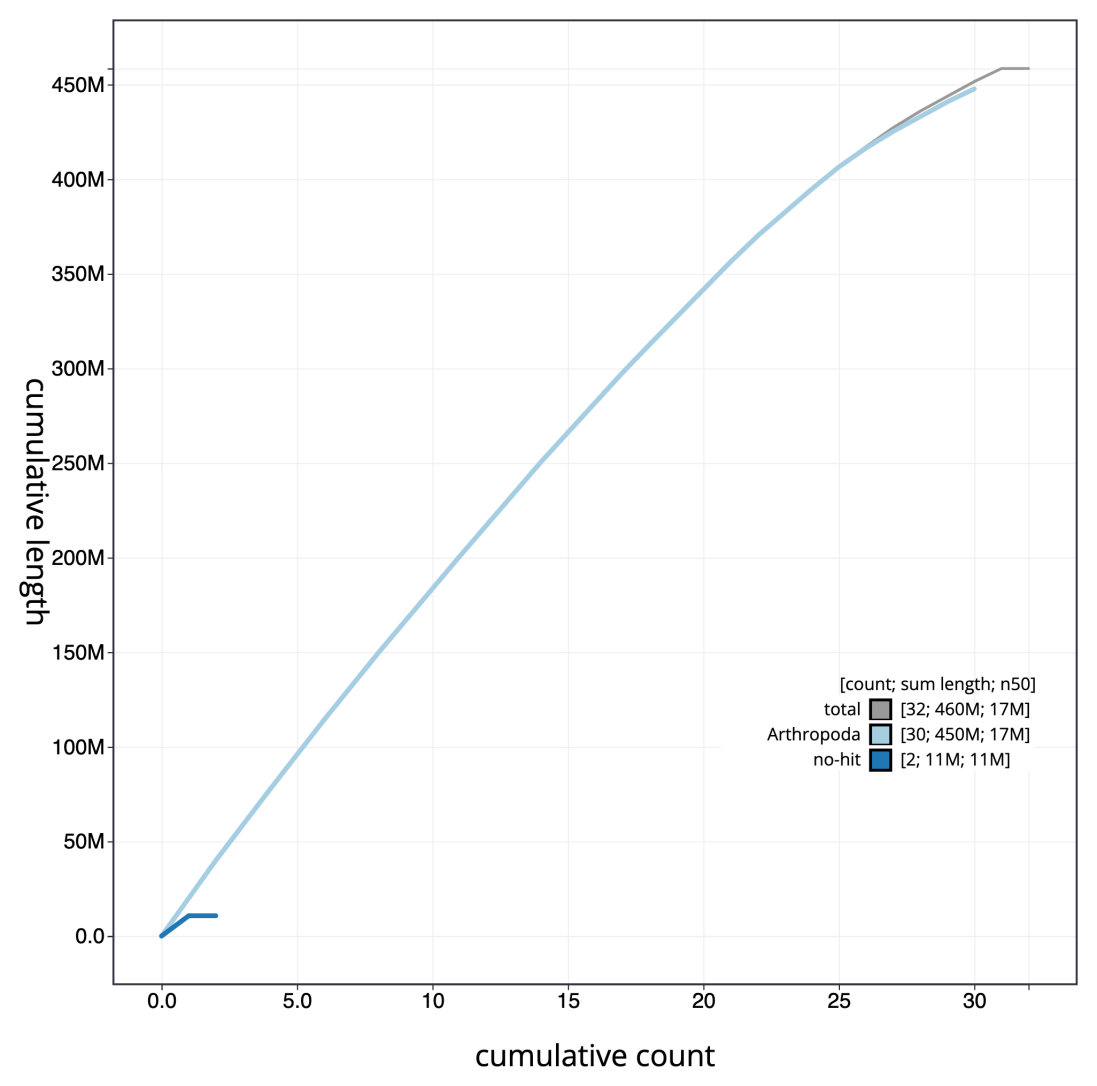
Genome assembly of *Allophyes oxyacanthae*, ilAllOxya1.1: cumulative sequence. BlobToolKit cumulative sequence plot. The grey line shows cumulative length for all scaffolds. Coloured lines show cumulative lengths of scaffolds assigned to each phylum using the buscogenes taxrule. An interactive version of this figure is available at https://blobtoolkit.genomehubs.org/view/ilAllOxya1_1/dataset/ilAllOxya1_1/cumulative.

**Figure 5.**
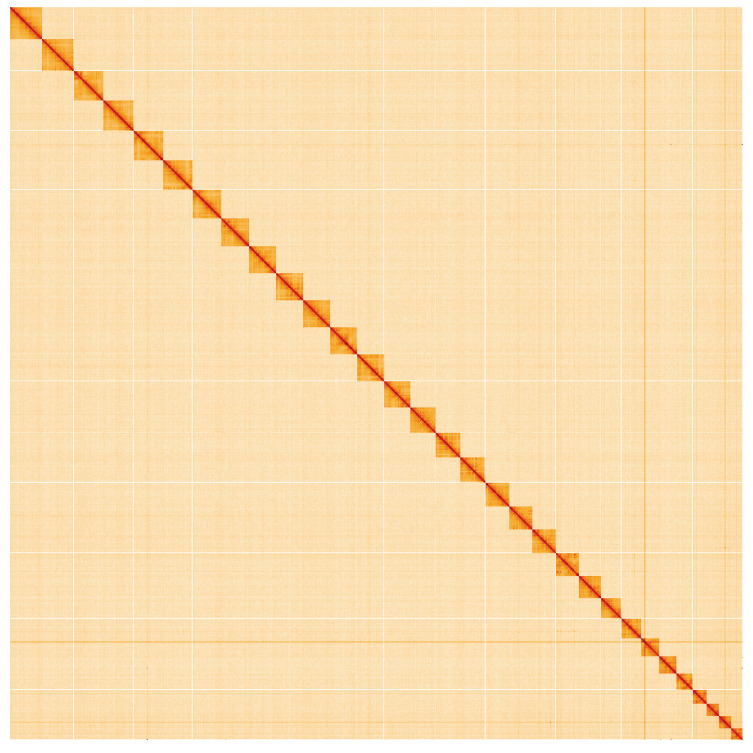
Genome assembly of *Allophyes oxyacanthae*, ilAllOxya1.1: Hi-C contact map. Hi-C contact map of the ilAllOxya1.1 assembly, visualised using HiGlass. Chromosomes are shown in order of size from left to right and top to bottom. An interactive version of this figure may be viewed at https://genome-note-higlass.tol.sanger.ac.uk/l/?d=OzgdewECSHeyOqN4f7ptEQ.

**Table 2.** Chromosomal pseudomolecules in the genome assembly of *Allophyes oxyacanthae*, ilAllOxya1.

INSDC accession	Chromosome	Size (Mb)	GC%
OW028610.1	1	19.67	36.3
OW028611.1	2	18.89	36.7
OW028612.1	3	18.72	36.7
OW028613.1	4	18.51	36.5
OW028614.1	5	18.2	36
OW028615.1	6	17.92	36.1
OW028616.1	7	17.47	36.2
OW028617.1	8	17.03	36.1
OW028618.1	9	17	35.9
OW028619.1	10	16.91	36.3
OW028620.1	11	16.8	36
OW028621.1	12	16.68	36.3
OW028622.1	13	16.39	36.2
OW028623.1	14	15.76	36.1
OW028624.1	15	15.71	36.1
OW028625.1	16	15.55	36.3
OW028626.1	17	14.93	36.5
OW028627.1	18	14.7	36.9
OW028628.1	19	14.63	36.5
OW028629.1	20	14.54	36.4
OW028630.1	21	13.74	36.1
OW028631.1	22	12.47	36.2
OW028632.1	23	12.29	36.7
OW028633.1	24	11.69	36.7
OW028634.1	25	10.72	36.3
OW028635.1	26	9.96	36.2
OW028636.1	27	8.76	36.6
OW028637.1	28	7.85	36.4
OW028638.1	29	7.81	37.7
OW028639.1	30	6.89	36.7
OW028609.1	Z	20.28	36.3
OW028640.1	MT	0.02	20.1

## Genome annotation report

The *A. oxyacanthae* genome assembly ( GCA_932294325.1) was annotated using the Ensembl rapid annotation pipeline ( Table 1). The resulting annotation includes 17,485 transcribed mRNAs from 17,301 protein-coding genes.

## Methods

### Sample acquisition and nucleic acid extraction

An individual male *A. oxyacanthae* specimen (ilAllOxya1) was collected in Wytham Woods, Oxfordshire (biological vice-county: Berkshire), UK (latitude 51.77, longitude –1.34) on 8 October 2020 using a light trap. The specimens were collected and identified by Douglas Boyes (University of Oxford) and snap-frozen on dry ice. This specimen was used for DNA and Hi-C sequencing.

DNA was extracted at the Tree of Life laboratory, Wellcome Sanger Institute (WSI). The ilAllOxya1 sample was weighed and dissected on dry ice with head tissue set aside for Hi-C sequencing. Thorax tissue was disrupted using a Nippi Powermasher fitted with a BioMasher pestle. High molecular weight (HMW) DNA was extracted using the Qiagen MagAttract HMW DNA extraction kit. HMW DNA was sheared into an average fragment size of 12–20 kb in a Megaruptor 3 system with speed setting 30. Sheared DNA was purified by solid-phase reversible immobilisation using AMPure PB beads with a 1.8X ratio of beads to sample to remove the shorter fragments and concentrate the DNA sample. The concentration of the sheared and purified DNA was assessed using a Nanodrop spectrophotometer and Qubit Fluorometer and Qubit dsDNA High Sensitivity Assay kit. Fragment size distribution was evaluated by running the sample on the FemtoPulse system.

### Sequencing

Pacific Biosciences HiFi circular consensus DNA sequencing libraries were constructed according to the manufacturers’ instructions. DNA sequencing was performed by the Scientific Operations core at the WSI on Pacific Biosciences SEQUEL II (HiFi) instrument. Hi-C data were also generated from head tissue of ilAllOxya1 using the Arima v2 kit and sequenced on the Illumina NovaSeq 6000 instrument.

### Genome assembly

Assembly was carried out with Hifiasm ( Cheng *et al.*, 2021) and haplotypic duplication was identified and removed with purge_dups ( Guan *et al.*, 2020). The assembly was scaffolded with Hi-C data ( Rao *et al.*, 2014) using YaHS ( Zhou *et al*., 2022). The assembly was checked for contamination as described previously ( Howe *et al.*, 2021). Manual curation was performed using HiGlass ( Kerpedjiev *et al.*, 2018) and Pretext ( Harry, 2022). The mitochondrial genome was assembled using MitoHiFi ( Uliano-Silva *et al.*, 2022), which performed annotation using MitoFinder ( Allio *et al.*, 2020). The genome was analysed and BUSCO scores generated within the BlobToolKit environment ( Challis *et al.*, 2020). Table 3 contains a list of all software tool versions used, where appropriate.

**Table 3.** Software tools and versions used.

Software tool	Version	Source
BlobToolKit	3.5.0	Challis *et al.*, 2020
Hifiasm	0.16.1-r375	Cheng *et al.*, 2021
HiGlass	1.11.6	Kerpedjiev *et al.*, 2018
MitoHiFi	2	Uliano-Silva *et al.*, 2022
PretextView	0.2	Harry, 2022
purge_dups	1.2.3	Guan *et al.*, 2020
YaHS	yahs- 1.1.91eebc2	Zhou *et al.*, 2022

### Genome annotation

The BRAKER2 pipeline ( Brůna *et al.*, 2021) was used in the default protein mode to generate annotation for the *Allophyes oxyacanthae* assembly (GCA_934047225.1) in Ensembl Rapid Release.

### Ethics/compliance issues

The materials that have contributed to this genome note have been supplied by a Darwin Tree of Life Partner. The submission of materials by a Darwin Tree of Life Partner is subject to the Darwin Tree of Life Project Sampling Code of Practice. By agreeing with and signing up to the Sampling Code of Practice, the Darwin Tree of Life Partner agrees they will meet the legal and ethical requirements and standards set out within this document in respect of all samples acquired for, and supplied to, the Darwin Tree of Life Project. Each transfer of samples is further undertaken according to a Research Collaboration Agreement or Material Transfer Agreement entered into by the Darwin Tree of Life Partner, Genome Research Limited (operating as the Wellcome Sanger Institute), and in some circumstances other Darwin Tree of Life collaborators.

## Data Availability

European Nucleotide Archive: *Allophyes oxyacanthae* (green-brindled crescent). Accession number PRJEB50741; https://identifiers.org/ena.embl/PRJEB50741. ( Wellcome Sanger Institute, 2022) The genome sequence is released openly for reuse. The *Allophyes oxyacanthae* genome sequencing initiative is part of the Darwin Tree of Life (DToL) project. All raw sequence data and the assembly have been deposited in INSDC databases. Raw data and assembly accession identifiers are reported in Table 1.

## Author information

Members of the University of Oxford and Wytham Woods Genome Acquisition Lab are listed here: https://doi.org/10.5281/zenodo.4789928.

Members of the Darwin Tree of Life Barcoding collective are listed here: https://doi.org/10.5281/zenodo.4893703.

Members of the Wellcome Sanger Institute Tree of Life programme are listed here: https://doi.org/10.5281/zenodo.4783585.

Members of Wellcome Sanger Institute Scientific Operations: DNA Pipelines collective are listed here: https://doi.org/10.5281/zenodo.4790455.

Members of the Tree of Life Core Informatics collective are listed here: https://doi.org/10.5281/zenodo.5013541.

Members of the Darwin Tree of Life Consortium are listed here: https://doi.org/10.5281/zenodo.4783558.
