# Supplementary material for: The genome sequence of the Green-brindled Crescent, Allophyes oxyacanthae (Linnaeus, 1758)
Source: Wellcome Open Res. 2023 Feb 3;8:53. [Version 1] doi: 10.12688/wellcomeopenres.18935.1 (PMC11325130; doi:10.12688/wellcomeopenres.18935.1)
Supplement: Peer review history

# Reviewer report: Reviewer response for version 1

**DOI:** 10.21956/wellcomeopenres.20993.r62177

**Published:** 2024-08-14

**Authors:** Min-jin Han

**Affiliations:** 1 State Key Laboratory of Silkworm Genome Biology, Institute of Sericulture and Systems Biology, Key Laboratory of Sericultural Biology and Genetic Breeding, Ministry of Agriculture and Rural Affairs, College of Sericulture, Textile and Biomass Sciences, Southwest University, Chongqing, China

The study completed the genome sequencing, assembly, and annotation of the Green-brindled Crescent, which provides a significant foundation for the biological research of the Green-brindled Crescent and comparative genomics research. However, the study report shows notable deficiencies in genome annotation, such as the annotation of repetitive sequences and protein-coding genes. The paper does not include annotation of repetitive sequences. In the annotation of protein-coding genes, there is no assessment of the accuracy and completeness of the predicted protein-coding genes. I have noticed that these issues seem to be common problems in genome studies of individual species in this journal.

Are sufficient details of methods and materials provided to allow replication by others?

Yes

Is the rationale for creating the dataset(s) clearly described?

Yes

Are the datasets clearly presented in a useable and accessible format?

Yes

Are the protocols appropriate and is the work technically sound?

Partly

Reviewer Expertise:

Genomics; Genetics and Evolution ; Insects

I confirm that I have read this submission and believe that I have an appropriate level of expertise to confirm that it is of an acceptable scientific standard.

# Reviewer report: Reviewer response for version 1

**DOI:** 10.21956/wellcomeopenres.20993.r63373

**Published:** 2023-11-14

**Authors:** Amali Thrimawithana

**Affiliations:** 1 Plant and Food Research Institute of New Zealand Ltd, Auckland, New Zealand

This article summarise the assembly and annotation of the Green-brindled Crescent, which is great to see. As it helps articulate the how the species colour polymorphism make work at a molecular level as well as add to the lepidopteran genome databases. 

 One thing I was keen to know was why is the colour variant is of importance? Though the background highlighted the need for it given currently low knowledge on the aspect, I failed to understand how the knowledge on the colour polymorphism might help? Would it be related to management of the species?

 Within the methods, I was wondering what sort of evidence was used to run the annotation through BRAKER pipeline? Did you have RNASeq data or protein data from similar species? This would be a useful information to add. If the data like RNASeq was generated by the project for this - it will need to be added to method section too.

 One other minor addition I thought might be useful is to mention the database used by Busco for completion checking of the genome - (I can see it in Figure 2, but thought would be good to have in the method section too).

 All in all, great to see more high quality lepidopteran genomes coming out - building great resource for lepidopteran studies.

Are sufficient details of methods and materials provided to allow replication by others?

Yes

Is the rationale for creating the dataset(s) clearly described?

Partly

Are the datasets clearly presented in a useable and accessible format?

Yes

Are the protocols appropriate and is the work technically sound?

Yes

Reviewer Expertise:

Bioinformatics - genomic/metagenomic/transcriptomics/comparative genomics

I confirm that I have read this submission and believe that I have an appropriate level of expertise to confirm that it is of an acceptable scientific standard, however I have significant reservations, as outlined above.

# Response

**Published:** 2024-03-21

**Authors:** Tree of Life Team Sanger

**Affiliations:** Wellcome Trust Sanger Institute, UK

Thank you for your constructive comments on this data note. Our responses to the reviewer's comments are given here:   

 1. **Comment: "**One thing I was keen to know was why is the colour variant is of importance?"

**Response: **In the **Background**, we note that colour variation may be advantageous in polluted areas, but research is not yet conclusive. Understanding the genetics behind this can help manage the species in such environments. Sequencing the genome of this and other species with colour variants will improve our knowledge of these traits, aiding in conservation efforts by identifying resilient genetic variants.  2. **Comment:**  "What sort of evidence was used to run the annotation through BRAKER pipeline?"

**Response:** The annotation of this genome is provided by Rapid Ensembl at the European Bioinformatics Institute, and is performed independently of the genome assembly and evaluation process presented here. Details of the BRAKER2 pipeline for EBI Ensembl Rapid are given here: https://rapid.ensembl.org/info/genome/genebuild/braker.html. 

 3. **Comment:** "One other minor addition I thought might be useful is to mention the database used by Busco for completion checking of the genome - (I can see it in Figure 2, but thought would be good to have in the method section too)."

**Response: **The BUSCO database is given in the **Genome sequence report:** "The assembly has a BUSCO v5.3.2 ( Manni et al., 2021) completeness of 99.2% (single 98.8%, duplicated 0.4%) using the lepidoptera_odb10 reference set ( *n* = 5286)." and is also given in Figure 2 and below Table 1.

# Reviewer report: Reviewer response for version 1

**DOI:** 10.21956/wellcomeopenres.20993.r63379

**Published:** 2023-08-07

**Authors:** William Walker III

**Affiliations:** 1 Temperate Tree Fruit and Vegetable Research Unit, USDA-ARS, Wapato, Washington, USA

The authors present a report on the genome sequence of the Green-brindled Crescent moth, *Allophyes oxyacanthae*. The manuscript is well written and straightforward, adhering to standards for this type of report, including information on predicted gene annotations. 

 This manuscript was previously examined by another reviewer, and I am inclined to agree with all comments of the previous reviewer, and would especially emphasize that I agree with the request for more detailed information on parameters/conditions for all of the bioinformatics software that were used. 

 Aside from that, I have no further comments, as the manuscript is otherwise very suitable for indexing.

Are sufficient details of methods and materials provided to allow replication by others?

No

Is the rationale for creating the dataset(s) clearly described?

Yes

Are the datasets clearly presented in a useable and accessible format?

Yes

Are the protocols appropriate and is the work technically sound?

Yes

Reviewer Expertise:

Insect genetics, genomics and molecular biology.

I confirm that I have read this submission and believe that I have an appropriate level of expertise to confirm that it is of an acceptable scientific standard, however I have significant reservations, as outlined above.

# Reviewer report: Reviewer response for version 1

**DOI:** 10.21956/wellcomeopenres.20993.r63371

**Published:** 2023-08-03

**Authors:** Lapo Ragionieri

**Affiliations:** 1 Institute of Zoology, University of Cologne, Cologne, Germany

The manuscript entitled “ *The genome sequence of the Green-brindled Crescent,* *Allophyes oxyacanthae (Linnaeus, 1758)*” is well written and all the information are clearly presented and the aim of the paper is to provide useful information to further studies genetic basis of color variations in the study species.

1. Is there any available information about genome size of the study species or sister species based on direct method such as FACS?
2. Can the authors provide detailed information how all programs were executed?
3. In table 3, is Braker2 missing?
4. Is there any accession number for the proteome?

Are sufficient details of methods and materials provided to allow replication by others?

No

Is the rationale for creating the dataset(s) clearly described?

Yes

Are the datasets clearly presented in a useable and accessible format?

Yes

Are the protocols appropriate and is the work technically sound?

Yes

Reviewer Expertise:

Genomics, transcriptomics and proteomics

I confirm that I have read this submission and believe that I have an appropriate level of expertise to confirm that it is of an acceptable scientific standard, however I have significant reservations, as outlined above.
